# Activating Cannabinoid receptor 2 alleviates iron overload-induced liver damage via dual modulation of STAT3/hepcidin and Nrf2/FPN1 pathways

**DOI:** 10.1186/s43556-026-00541-1

**Published:** 2026-08-10

**Authors:** Han Deng, Yuqian Ren, Yunjie Sui, Zegang Ma

**Affiliations:** 1 Department of Physiology, School of Basic Medicine, Qingdao University, Room 507, Boya Building, 308 Ningxia Road, Qingdao, 266071 China; 2 Institute of Brain Science and Disorders, Qingdao University, Qingdao, China

**Keywords:** Cannabinoid receptor 2, Iron overload, Kupffer cells, Hepcidin, Ferroportin 1

## Abstract

Iron overload-related liver injury is a major clinical problem in hereditary hemochromatosis, transfusional siderosis, and chronic liver diseases, yet current treatments such as iron chelation therapy and phlebotomy are limited by safety and efficacy issues. Cannabinoid receptor type 2 (CB2R) has been implicated in the regulation of iron metabolism, but its role and underlying mechanism in iron overload remain unclear. Here, we investigated whether CB2R can prevent liver damage caused by iron overload and explored its potential mechanism. Male C57BL/6J mice received 100 mg/kg iron-dextran intraperitoneally to establish an iron overload model. Liver damage and iron deposition were evaluated via histology, serum biochemistry, and molecular analyses. Further studies using primary hepatocytes and Kupffer cells exposed to ferric ammonium citrate (FAC) were performed to explore cell type-specific mechanisms in vitro. Pharmacological CB2R activation with the selective agonist JWH133 reduced liver iron deposition, oxidative stress, and inflammation, whereas CB2R-knockout (KO) exacerbated these phenotypes. Mechanistically, CB2R activation suppressed signal transducer and activator of transcription 3 (STAT3)-dependent hepcidin transcription in hepatocytes, thereby enhancing ferroportin 1 (FPN1)-mediated iron efflux. Concurrently, CB2R promoted nuclear factor erythroid 2-related factor 2 (Nrf2)/FPN1 signaling in Kupffer cells, decreasing the labile iron pool. These findings identify CB2R as a key regulator of hepatic iron homeostasis and suggest that targeting CB2R may offer a novel therapeutic strategy for iron overload-related diseases.

**Supplementary Information:**

The online version contains supplementary material available at 10.1186/s43556-026-00541-1.

## Introduction

Iron is indispensable for oxygen transport and enzymatic catalysis, yet its excessive accumulation triggers oxidative tissue damage through Fenton reaction-derived reactive oxygen species (ROS) [1, 2]. Clinically, iron overload occurs in hereditary hemochromatosis, transfusional siderosis, chronic liver diseases, and in patients receiving long-term parenteral iron supplementation [3]. The liver, as the primary organ for iron storage, is particularly vulnerable to overload-associated injury [1, 4]. Excessive iron deposition in the liver disrupts redox balance, promotes lipid peroxidation, and activates pro-inflammatory pathways, ultimately causing progressive liver damage [3, 5, 6]. Current primary treatments, including iron chelation and phlebotomy [2], are associated with adverse effects, underscoring the need for novel therapeutic targets.

Emerging evidence implicates cannabinoid receptor type 2 (CB2R), a G protein-coupled receptor known for its anti-oxidative and anti-inflammatory properties, as a crucial modulator in various pathological states [7, 8]. Notably, CB2R is expressed in the liver and prior work has highlighted its hepatoprotective effects across various injury models, including nonalcoholic steatohepatitis and toxin-induced hepatotoxicity [9–13]. These protective mechanisms typically involve the suppression of inflammatory cascades and the restoration of cellular redox homeostasis, suggesting that CB2R activation could serve as an effective strategy against liver damage.

Recently, CB2R has also been identified as a potential modulator of iron metabolism. Seminal studies by *Seo* et al. revealed that CB2R activation inhibited the function of divalent metal transporter 1 (DMT1) in HEK293T cells by preventing its serine 43 phosphorylation [14]. Our prior work further demonstrated that the selective CB2R agonist JWH133 significantly inhibited iron influx into astrocytes, reduced iron deposition in the substantia nigra (SN), and regulated iron metabolic proteins in Parkinson’s disease (PD) models [8, 15]. Moreover, CB2R activation promotes iron transfer out of osteoclasts by regulating proteins DMT1 and ferroportin 1 (FPN1) proteins in inflammatory bowel disease (IBD) patients [16]. Further studies confirmed that CB2R regulates iron metabolism in peripheral IBD macrophages via the hepcidin/FPN1 axis [17]. Despite these findings suggesting a conserved role for CB2R in systemic iron regulation, whether CB2R protects against liver iron overload and how it specifically regulates hepatic iron metabolism remains largely unexplored.

Therefore, the objective of the present study was to determine whether CB2R protects against iron overload-induced liver injury and to elucidate its underlying in vivo and in vitro mechanisms. We utilized iron-dextran-treated wild-type (WT) and CB2R-knockout (KO) mice to evaluate hepatic injury and iron deposition, alongside primary cultures of hepatocytes and Kupffer cells exposed to ferric ammonium citrate (FAC) to explore cell type-specific mechanisms. Our study demonstrated that pharmacological CB2R activation significantly alleviated liver injury, oxidative stress, and iron overload, whereas its genetic deficiency aggravated these toxic phenotypes. Mechanistically, we discovered two complementary pathways: CB2R inhibited STAT3-dependent hepcidin transcription in hepatocytes while concurrently enhancing Nrf2/FPN1-mediated iron efflux in Kupffer cells. These results suggested that CB2R may be a key regulator of liver iron metabolism and a potential therapeutic target for iron overload-related liver diseases.

## Results

### Pharmacological CB2R activation mitigates iron overload-induced liver injury, oxidative stress and inflammation

**Fig. 1:**
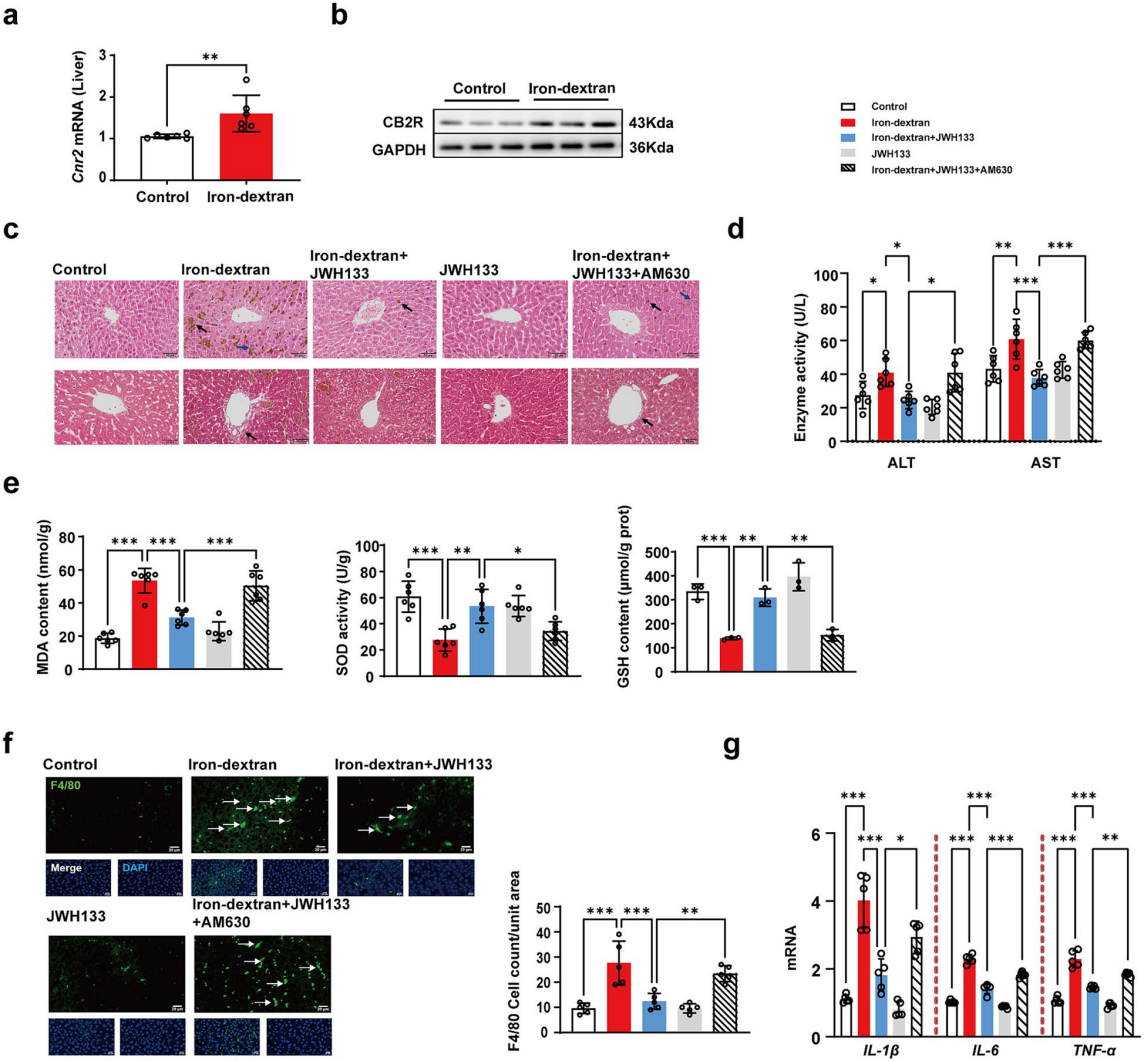
Pharmacological CB2R activation mitigates iron overload-induced liver injury, oxidative stress, and inflammation in vivo. **a** Hepatic *Cnr2* mRNA levels determined by qRT-PCR (*n* = 6). **b** Representative Western blot of CB2R protein in the liver, with GAPDH as the loading control. **c** Representative images of H&E staining (black arrows indicate iron deposition; blue arrows indicate inflammatory cell infiltration) and Masson staining (scale bar = 50 µm). **d** Serum ALT and AST activities across all experimental groups (*n* = 6). **e** Liver MDA content, SOD activity, and GSH levels indicating oxidative stress and antioxidant status (*n* = 6, 6, 3 respectively). **f** Representative immunofluorescence staining for the macrophage marker F4/80 (green) with DAPI counterstain (blue, scale bar = 20 µm) and corresponding quantification of F4/80 positive cells per unit area (*n* = 5). **g** Relative hepatic mRNA levels of pro-inflammatory cytokines *IL-1β*, *IL-6*, and *TNF-α* (*n* = 5). ^*^P < 0.05, ^**^P < 0.01, ^***^P < 0.001. Data are presented as the mean ± SD for each group. Individual data points are displayed as dots

To determine whether CB2R is involved in the hepatic response to iron overload, we first examined its expression. Iron-dextran challenge significantly upregulated hepatic CB2R mRNA (*Cnr2*) and protein levels compared to controls (Fig. 1a-b). Iron overload often leads to liver injury [2]. We next assessed whether pharmacological CB2R activation mitigates iron-induced liver injury. Hematoxylin and eosin (H&E) staining revealed marked brownish-yellow hemosiderin deposition and inflammatory cell infiltration in iron-overloaded livers, while Masson staining showed pronounced collagen accumulation (Fig. 1c). Pretreatment with the selective CB2R agonist JWH133 significantly attenuated these histopathological abnormalities and reduced serum ALT/AST elevations, whereas co-administration of the CB2R antagonist AM630 largely reversed these protective effects (Fig. 1d). Because iron overload is known to trigger oxidative stress, we next quantified lipid peroxidation and antioxidant defenses [18]. As shown in the Fig. 1e, the malondialdehyde (MDA) content increased by approximately 190% in the iron-dextran group compared with control, whereas superoxide dismutase (SOD) activity and glutathione (GSH) levels decreased by about 55% and 58%, respectively. JWH133 treatment significantly attenuated lipid peroxidation and restored antioxidant capacity. Furthermore, immunofluorescence for macrophage marker F4/80 and qRT-PCR analysis revealed that iron overload triggered robust macrophage accumulation and pro-inflammatory cytokine transcription (*IL-1β*, *IL-6*, and *TNF-α*), which were effectively blunted by JWH133 (Fig. 1f-g).

Collectively, CB2R expression is upregulated in the liver under iron overload, and pharmacological CB2R activation effectively alleviates iron overload-induced liver injury, oxidative stress, and inflammation.

### CB2R deficiency exacerbates iron overload-induced liver injury, oxidative stress, and inflammation

**Fig. 2:**
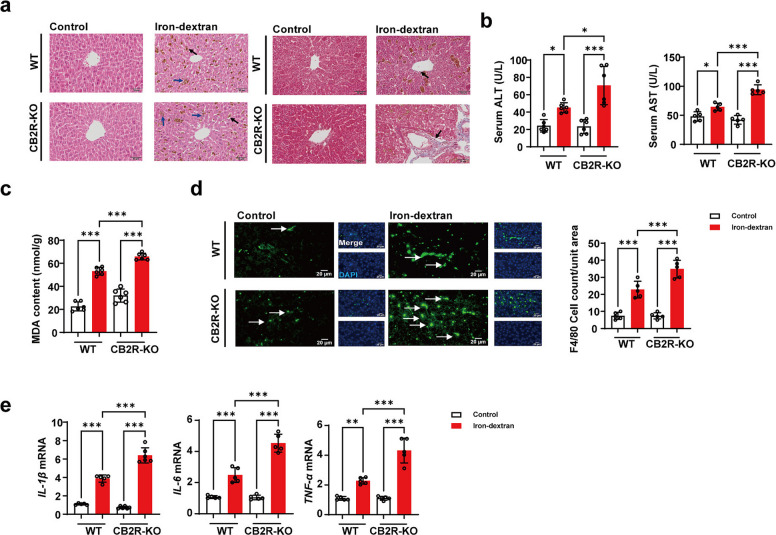
CB2R deficiency exacerbates iron overload-induced liver injury and inflammation. **a** Representative H&E and Masson staining of liver sections from WT and CB2R-KO mice (scale bar = 50 µm). **b** Serum ALT and AST activities (*n* = 6, 5 respectively). **c** Liver MDA content (*n* = 6). **d** Representative immunofluorescence staining for F4/80 (scale bar = 20 µm) and corresponding quantification of F4/80 positive cells in WT and CB2R-KO mice (*n* = 5). **e** Relative hepatic mRNA levels of *IL-1β*, *IL-6*, and *TNF-α* (*n* = 5). ^*^P < 0.05, ^**^P < 0.01, ^***^P < 0.001. Data are presented as the mean ± SD for each group. Individual data points are displayed as dots

To clarify the protective role of endogenous CB2R, we next compared the responses of WT and CB2R-KO mice to iron-dextran. As expected, the results opposed those observed with JWH133 treatment. Histopathological analysis revealed that CB2R deficiency markedly exacerbated iron-dextran–induced liver damage, with CB2R-KO mice displaying more severe inflammatory infiltration, iron deposition, and hepatic fibrosis compared with iron-overloaded WT mice (Fig. 2a). This aggravated histology was accompanied by a further deterioration in liver function, with serum ALT and AST levels significantly higher in CB2R-KO mice than in WT controls (Fig. 2b). Furthermore, CB2R-KO enhanced lipid peroxidation, resulting in elevated MDA content (Fig. 2c). The inflammatory response was concurrently intensified, evidenced by increased macrophage infiltration and profoundly heightened mRNA expressions of *IL-1β*, *IL-6*, and *TNF-α* compared to WT iron-overloaded mice (Fig. 2d-e). These findings indicate that CB2R deficiency makes the liver more susceptible to iron overload, which leads to increased liver tissue damage, oxidative stress, and inflammation.

### Pharmacological CB2R activation limits hepatic iron accumulation by modulating the hepcidin-FPN1 axis

**Fig. 3:**
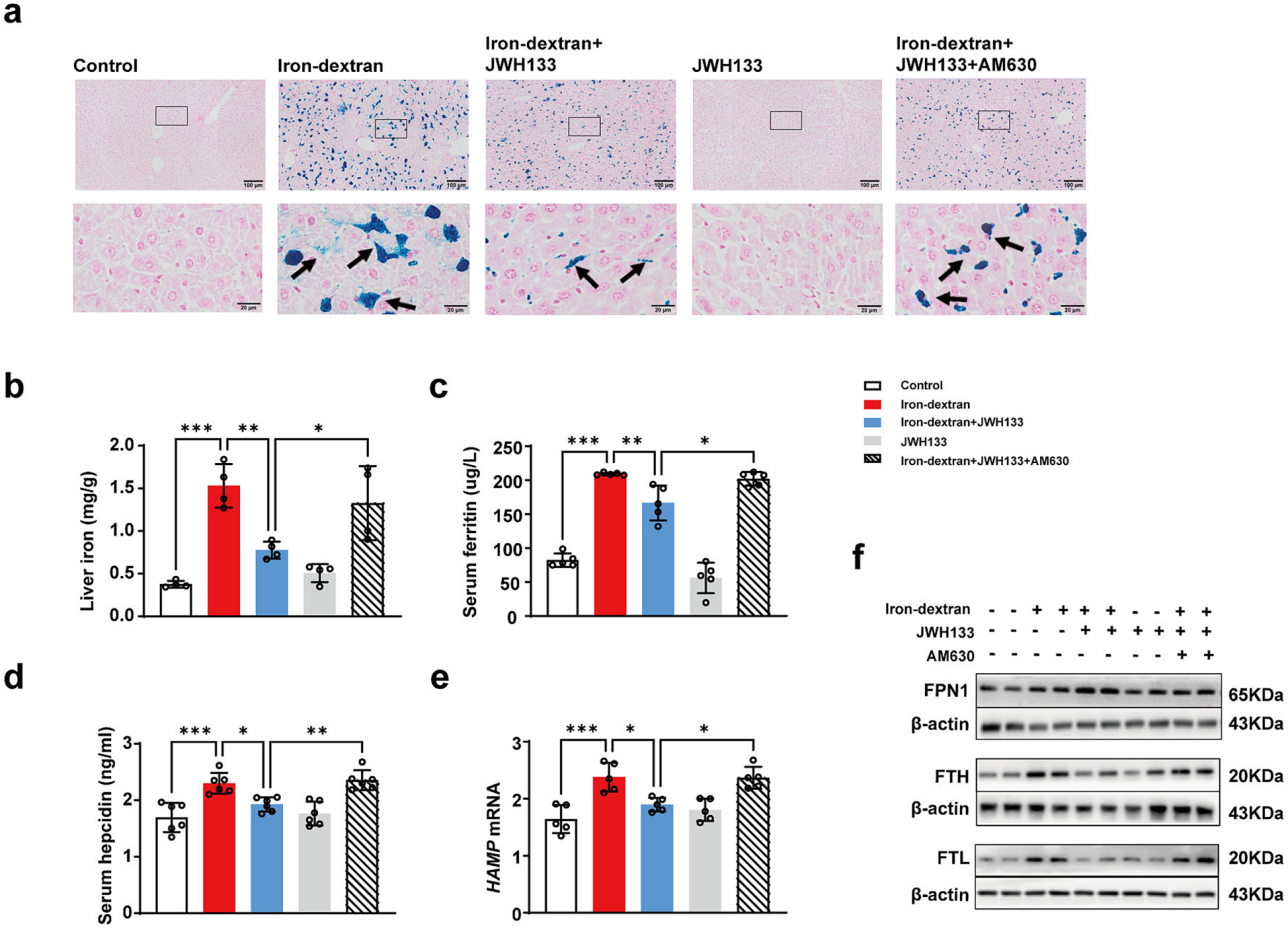
Pharmacological CB2R activation limits hepatic iron accumulation by modulating the hepcidin-FPN1 axis. **a** Representative Prussian blue staining of liver sections from pharmacological treatment groups (black arrows indicate positive blue iron deposits; scale bars = 100 and 20 µm). **b** Liver iron concentrations quantified using a ferrozine-based assay (*n* = 4). **c** Serum ferritin levels determined using ELISA (*n* = 5). **d** Serum hepcidin levels using ELISA (*n* = 6). **e** Hepatic *HAMP* mRNA expression determined by qRT-PCR (*n* = 5). **f** Representative Western blots of iron-related proteins FPN1, FTH, and FTL in the liver across different treatment groups, with β-actin as the loading control. ^*^P < 0.05, ^**^P < 0.01, ^***^P < 0.001. Data are presented as the mean ± SD for each group. Individual data points are displayed as dots

Given that CB2R-KO mice were more sensitive to iron-induced hepatotoxicity, we next examined whether CB2R regulates hepatic iron accumulation under iron overload conditions. Prussian blue staining confirmed that JWH133 visibly limited liver iron deposition (Fig. 3a). Pharmacological activation of CB2R with JWH133 significantly attenuated iron-dextran–induced iron accumulation, reducing liver iron content by 49% and serum ferritin by 20% relative to the iron-dextran group, whereas co-administration of the CB2R antagonist AM630 largely abolished these effects (Fig. 3b-c). The liver secretes hepcidin (*HAMP*), which regulates iron homeostasis by binding to the iron exporter FPN1 [1, 19, 20]. We then explored whether the hepcidin-FPN1 axis mediates the CB2R-dependent regulation of hepatic iron. Iron challenge prominently induced serum hepcidin and *HAMP* mRNA. JWH133 successfully counteracted this hepcidin induction (Fig. 3d-e). Western blot analysis revealed that JWH133 further enhanced the expression of the iron exporter FPN1, while simultaneously reducing the iron storage proteins FTH and FTL relative to the iron-dextran group (Fig. 3f). Collectively, these data suggest that CB2R activation promotes iron efflux and limits hepatic iron storage by suppressing hepcidin and coordinately increasing FPN1 while decreasing ferritin, thereby alleviating liver iron overload in vivo.

### CB2R deficiency aggravates iron accumulation while its overexpression alleviates accumulation

**Fig. 4:**
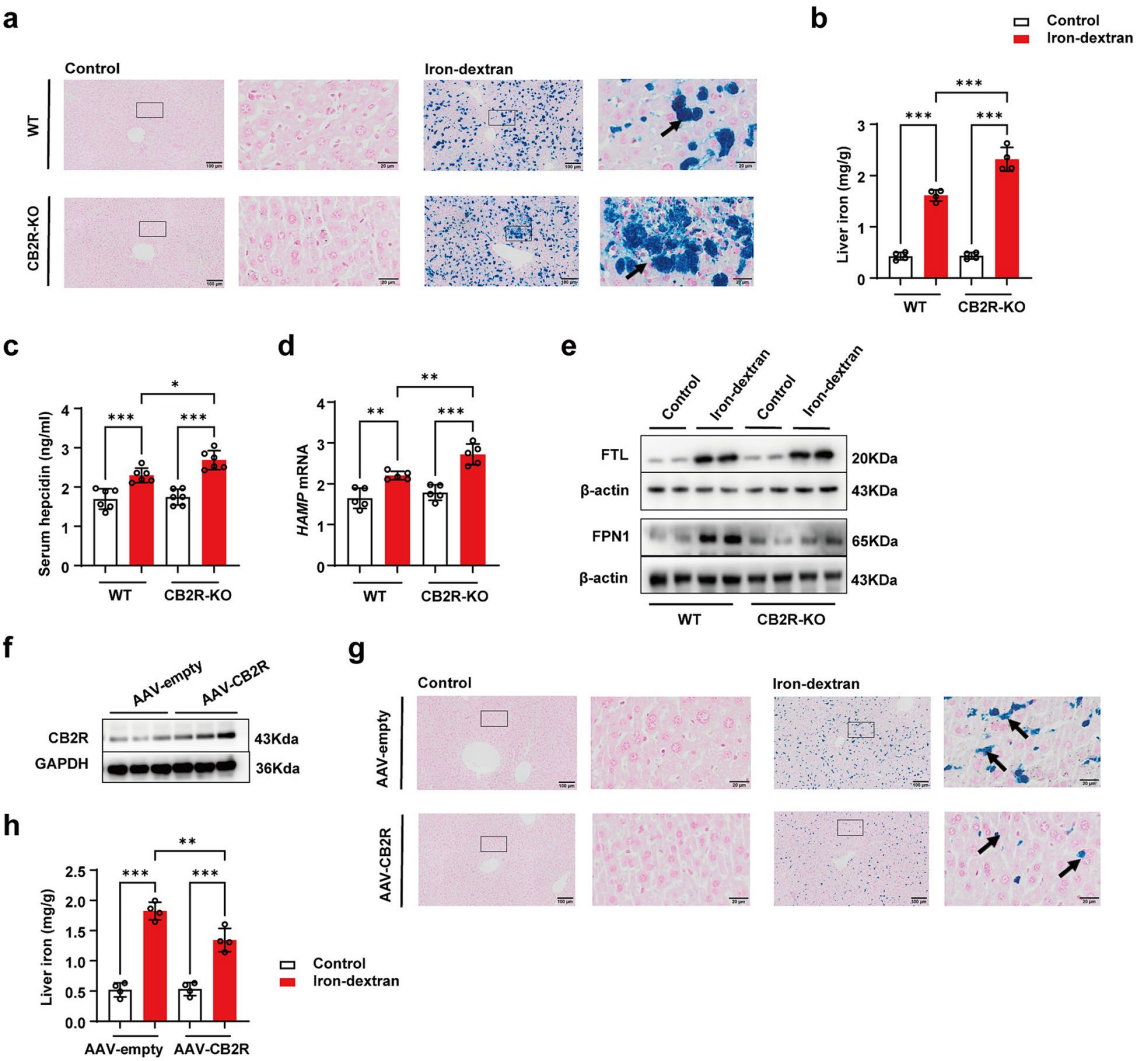
CB2R deficiency aggravates iron retention while its overexpression alleviates accumulation. **a** Representative Prussian blue staining of liver sections from WT and CB2R-KO mice (scale bars = 100 and 20 µm). **b** Liver iron concentrations in WT and CB2R-KO mice (*n* = 4). **c** Serum hepcidin levels (*n* = 6). **d** Hepatic *HAMP* mRNA expression (*n* = 5). **e** Representative Western blots of FTL and FPN1 in the liver of WT and CB2R-KO mice, with β-actin as the loading control. **f** Representative Western blot verifying AAV-mediated CB2R overexpression in the liver, with GAPDH as the loading control. **g** Prussian blue staining of liver sections from AAV-empty and AAV-CB2R injected mice. **h** Liver iron concentrations following AAV injection (*n* = 4). ^*^P < 0.05, ^**^P < 0.01, ^***^P < 0.001. Data are presented as the mean ± SD for each group. Individual data points are displayed as dots

To corroborate the pharmacological findings, we examined iron homeostasis genetically. Prussian blue staining and ferrozine assays revealed a more widespread iron accumulation in CB2R-KO mice, with liver iron content 44% higher than in iron-treated WT mice (Fig. 4a-b). At the molecular level, CB2R-KO dramatically amplified hepcidin expression during iron overload, resulting in decreased FPN1 and increased FTL protein levels compared to WT counterparts (Fig. 4c-e). Finally, to establish sufficiency, an adeno-associated virus (AAV) was utilized to overexpress CB2R specifically in the liver (Fig. 4f). Following iron challenge, AAV-CB2R markedly reduced Prussian blue-positive iron deposition and significantly decreased hepatic iron content by approximately 30% compared to AAV-empty controls (Fig. 4g-h). In summary, both genetic loss- and gain-of-function evidence firmly establishes CB2R as a pivotal negative regulator of hepatic iron accumulation via the hepcidin-FPN1 axis.

### CB2R protects against iron-induced toxicity in hepatocytes by suppressing the STAT3/hepcidin pathway

**Fig. 5:**
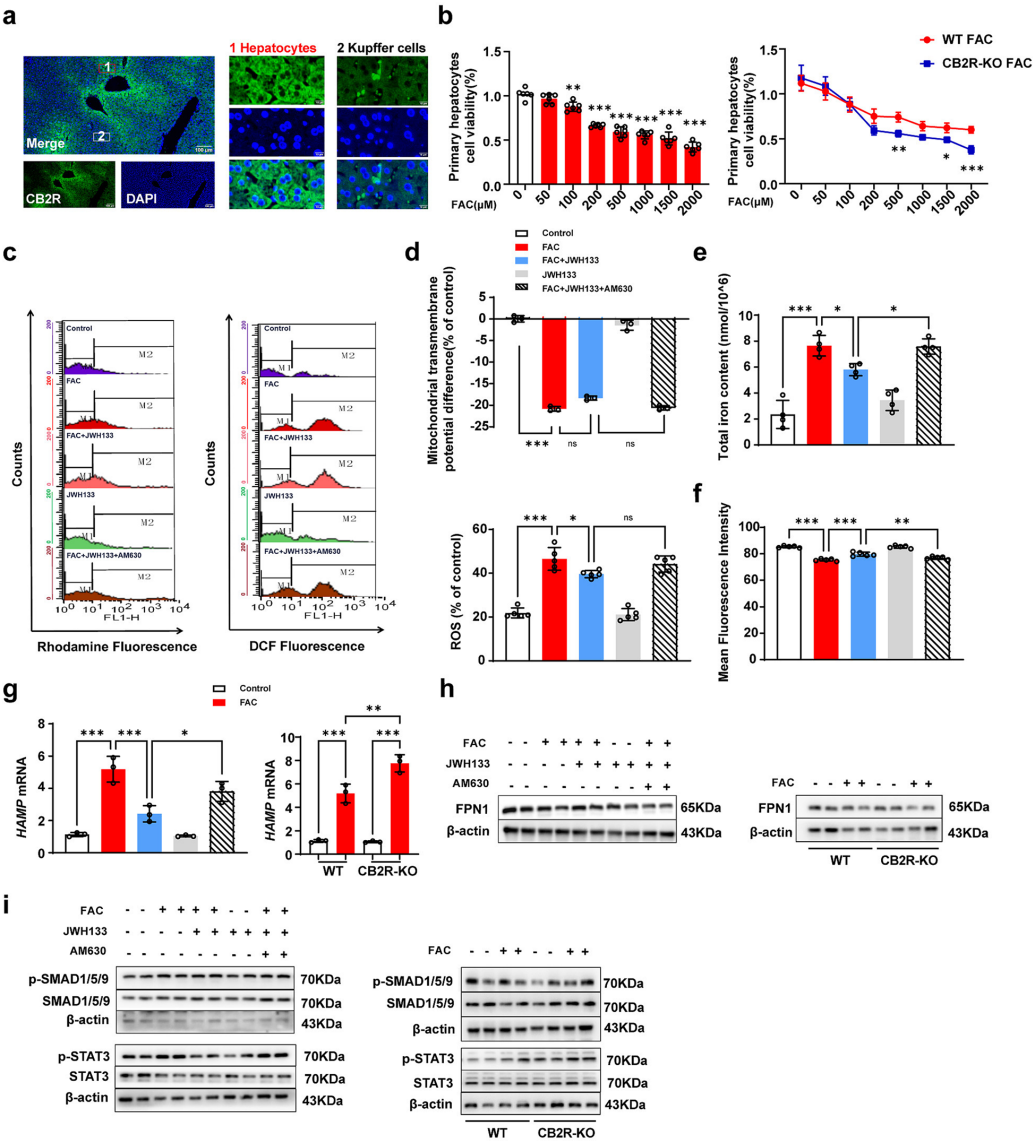
CB2R protects against iron-induced toxicity in hepatocytes by suppressing the STAT3/hepcidin pathway. **a** Immunofluorescence detection of CB2R localisation in liver (green). Nuclear counterstaining was performed with DAPI (blue, Scar bar = 100 and 20 μm). **b** Cell viability measured by CCK-8 assay across different concentrations of FAC, and comparison between WT and CB2R-KO hepatocytes (*n* = 6, 6 respectively). **c** Representatives of the flurometric assay on MMP and ROS in different treatment groups. **d** Summarized data demonstrated changes in MMP and ROS in hepatocytes (*n* = 3, 5 respectively). **e** Total cellular iron content (*n* = 4). **f** The level of LIP was detected by using flow cytometry (*n* = 5). **g** *HAMP* mRNA levels determined by qRT-PCR (*n* = 3, 3 respectively). **h** Representative Western blots of FPN1 protein, with β-actin as the loading control. **i** Representative Western blots of the upstream regulatory signaling molecules p-SMAD1/5/9, SMAD1/5/9, p-STAT3, and STAT3, with β-actin as the loading control. ^*^P < 0.05, ^**^P < 0.01, ^***^P < 0.001. Data are presented as the mean ± SD for each group. Individual data points are displayed as dots

While initial studies suggested CB2R is primarily expressed in Kupffer cells, recent evidence indicates it is also expressed in hepatocytes [9, 21–23]. Immunofluorescence confirmed CB2R was expressed in both hepatocytes and Kupffer cells (Fig. 5a). Exposure to different concentrations of ferric ammonium citrate (FAC) reduced WT hepatocyte viability, whereas CB2R-KO hepatocytes exhibited heightened vulnerability to severe iron toxicity (Fig. 5b). Flow-cytometric analysis revealed that FAC markedly decreased mitochondrial membrane potential (MMP) and increased intracellular ROS, whereas JWH133 pretreatment inhibited intracellular ROS production (Fig. 5c-d). Consistently, FAC exposure increased total cellular iron and enlarged the labile iron pool (LIP). JWH133 pretreatment significantly decreased total iron content by roughly 20% and partially restored calcein fluorescence, indicating a reduction in LIP, whereas AM630 co-treatment blunted these effects (Fig. 5e-f). Hepatocytes are the major cells that synthesize and secrete hepcidin [4]. Mechanistically, FAC heavily induced *HAMP* mRNA in WT hepatocytes. JWH133 inhibited this induction while upregulating FPN1 protein (Fig. 5g-h). In contrast, CB2R-KO hepatocytes exhibited profoundly exaggerated *HAMP* induction without enhancing FPN1 (Fig. 5g-h). Bone morphogenetic protein (BMP)/drosophila mothers against decapentaplegic protein (SMAD) and IL-6/Janus kinase-2 (JAK2)/STAT3 are two classic signaling pathways that regulate hepcidin, we next assessed these signaling cascades [19, 20, 24]. JWH133 markedly suppressed FAC-induced STAT3 phosphorylation without altering SMAD signaling, whereas CB2R-KO enhanced STAT3 phosphorylation (Fig. 5i). Consequently, CB2R protects hepatocytes from iron toxicity by selectively inhibiting STAT3 phosphorylation, which blunts hepcidin transcription and restores FPN1-mediated iron export.

### CB2R mitigates iron-induced injury in Kupffer cells by promoting Nrf2/FPN1-mediated iron export

**Fig. 6:**
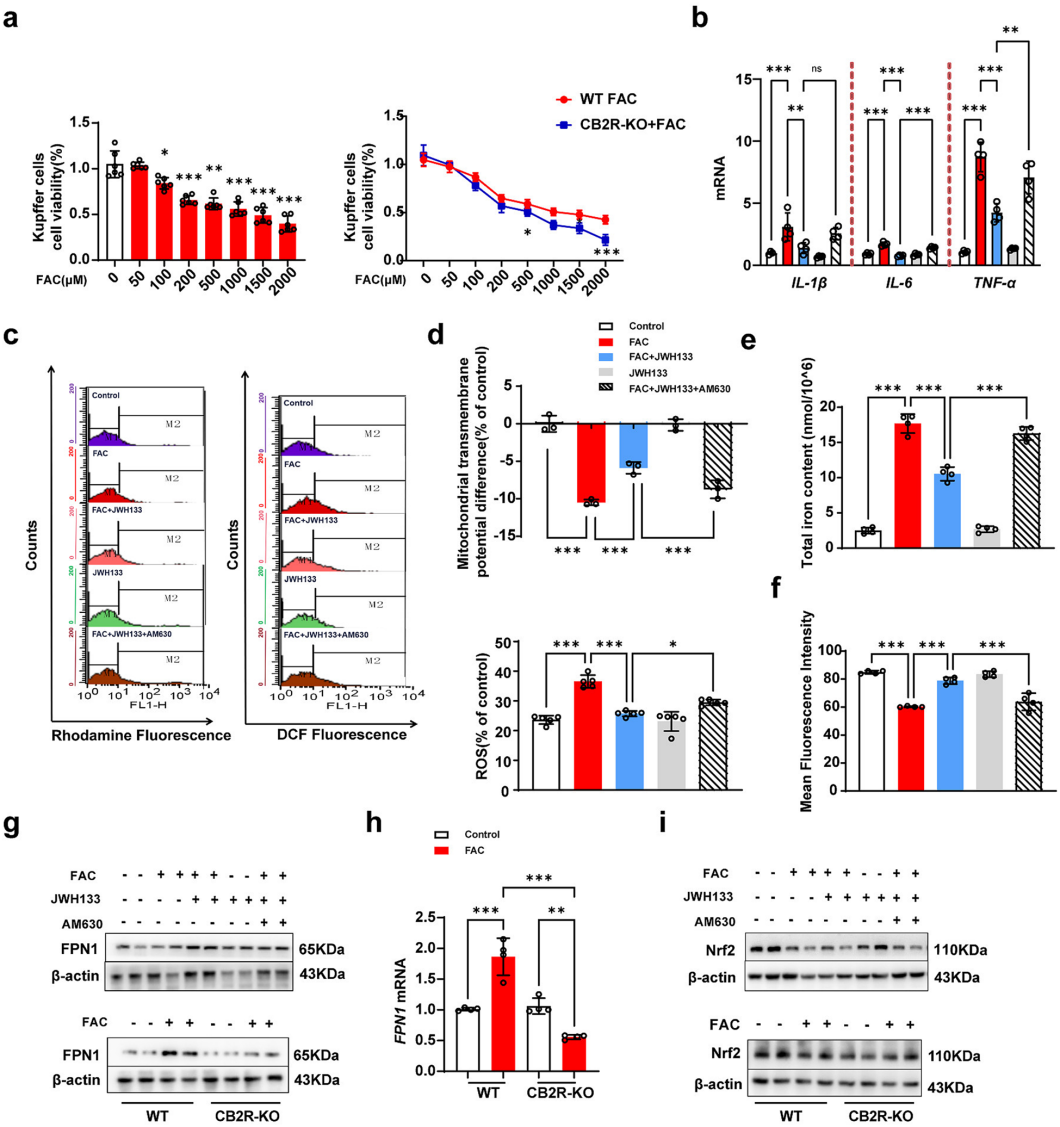
CB2R mitigates iron-induced injury in Kupffer cells by promoting Nrf2/FPN1-mediated iron export. **a** Cell viability of WT and CB2R-KO Kupffer cells exposed to FAC, measured by CCK-8 assay (*n* = 6, 6 respectively). **b** Relative mRNA levels of pro-inflammatory cytokines *IL-1β*, *IL-6*, and *TNF-α* (*n* = 4, 4, 4 respectively). **c** Representatives of the flurometric assay on MMP and ROS in different treatment groups. **d** Summarized data demonstrated changes in MMP and ROS in kupffer cells (*n* = 3, 5 respectively). **e** Total cellular iron content (*n* = 4). **f** The level of LIP was detected by using flow cytometry (*n* = 4). **g** Representative Western blots of FPN1 protein in different treatment groups, with β-actin as the loading control. **h** *FPN1* mRNA expression levels determined by qRT-PCR (*n* = 4). **i** Representative Western blots of Nrf2 protein in different treatment groups, with β-actin as the loading control. ^*^P < 0.05, ^**^P < 0.01, ^***^P < 0.001. Data are presented as the mean ± SD for each group. Individual data points are displayed as dots

Macrophages can engulf senescent red blood cells, break them down to produce iron, and subsequently export iron through the FPN1-dependent pathway [20]. The macrophages in the liver are Kupffer cells, which work together with hepatocytes to regulate iron metabolism [25–27]. Similar to hepatocytes, CB2R-KO Kupffer cells showed greater vulnerability to severe iron overload (Fig. 6a). In primary Kupffer cells, FAC induced robust transcription of pro-inflammatory cytokines (*IL-1β*, *IL-6* and *TNF-α*), which was significantly dampened by JWH133 (Fig. 6b). JWH133 concurrently restored MMP, inhibited ROS generation, and drastically reduced both total cellular iron and LIP levels (Fig. 6c-f). As the key protein known to mediate iron export, FPN1 facilitates iron efflux from Kupffer cells [28]. As shown in Fig. 6g, JWH133 significantly increased the expression level of FPN1 protein compared with FAC alone. In CB2R KO cells, both FPN1 protein and mRNA expression decreased after FAC treatment, suggesting that CB2R regulates FPN1 at least in part at the transcriptional level (Fig. 6g-h). Given that the nuclear factor Nrf2 has been reported to regulate *FPN1* transcription [29, 30], we next assessed Nrf2 protein levels. We found that JWH133 elevated Nrf2 protein expression in FAC-treated WT cells, whereas Nrf2 levels in CB2R-KO cells were substantially lower than in WT controls (Fig. 6i). Altogether, these results reveal that CB2R activation enhances the Nrf2/FPN1 signaling axis in Kupffer cells to promote iron efflux, whereas CB2R deficiency weakens this pathway and increases the susceptibility of Kupffer cells to iron overload.

## Discussion

Systemic iron overload emerges when physiological iron retention exceeds the body’s metabolic capacity, typically driven by metabolic dysregulation, specific genetic disorders, or excessive external supplementation [2, 31–33]. If left unmanaged, this surplus iron drives widespread tissue toxicity and severe clinical morbidities. Given its central role as the primary reservoir for systemic iron, the liver is highly susceptible to such progressive damage [2, 34]. Clinically, long-term or inappropriate administration of iron preparations such as iron-dextran can induce hepatic iron deposition and contribute to steatohepatitis, fibrosis, and cirrhosis [22]. In this study, we successfully established an iron overload model in mice by intraperitoneal injection 100 mg/kg iron-dextran. Previous studies have confirmed that CB2R activation exerts hepatoprotective effects via anti-oxidative, anti-inflammatory, and anti-apoptotic mechanisms in several models of liver injury [21, 22, 35]. However, there are no reports on the therapeutic strategies targeting CB2R to reduce liver injury caused by iron overload. This study is the first to explore the role of CB2R in a mouse model of iron overload, and the molecular mechanisms were investigated.

In this study, the excessive accumulation of iron in the iron-overloaded model mice led to liver injury. Meanwhile, iron overload could increase the expression of CB2R in the liver. Iron overload-induced oxidative stress and the subsequent release of pro-inflammatory cytokines may account for the increase of CB2R in the liver, as pathological stress and inflammatory stimuli are potent inducers of CB2R expression [7, 36, 37]. Indeed, CB2R has been shown to be upregulated in various chronic liver diseases, such as non-alcoholic fatty liver disease and cirrhosis [38, 39]. Moreover, under pathological conditions such as Alzheimer’s disease, multiple sclerosis, stroke, and neuropathic pain, the upregulation of CB2R expression in microglia and neurons has also been confirmed [37, 40, 41]. Overall, CB2R is upregulated in these pathological tissues or cells, potentially serving as an endogenous compensatory mechanism to mitigate the resulting damage. Our study found that CB2R activation by JWH133 could alleviate liver injury, oxidative stress and inflammation caused by iron overload. Furthermore, liver injury induced by iron overload was aggravated in CB2R-KO mice. These results support the elevation of CB2R in iron overload as an endogenous protective mechanism. Activation of CB2R can protect liver injury caused by iron overload. At the same time, our data do not exclude the possibility that in other disease settings or at different stages, CB2R upregulation could become insufficient or dysregulated, an issue that warrants further temporal and dose–response studies.

Previous studies have found that activation of CB2R can regulate iron metabolism-related proteins to reduce intracellular iron content in PD, IBD and other diseases that cause iron overload [10, 15, 17]. This study also supported the regulatory effect of CB2R on liver iron metabolism is closely related to a variety of key proteins and signaling pathways. Hepcidin, as a core peptide hormone regulating iron metabolism, can bind to membrane iron transporter (FPN1), promote its degradation, and thereby reduce intracellular iron output [1, 19, 20]. Hepcidin is normally upregulated during iron overload, thereby blocking iron export and favoring hepatic iron retention [42]. Here, CB2R activation inhibited hepcidin expression while upregulating FPN1 expression to promote iron excretion and effectively alleviate liver iron overload. Ferritin is the main intracellular iron storage protein and is composed of heavy (FTH) and light (FTL) subunits [2, 43]. By buffering labile iron, ferritin protects against iron-catalyzed ROS formation but also reflects increased iron storage [2]. In our study, CB2R activation reduced FTH and FTL expression, thereby limiting liver iron storage. However, there is currently no direct evidence that CB2R signaling targets the ferritin gene or the protein itself. Combined with previous studies and the present study, we hypothesized that CB2R reduced intracellular iron utilization and indirectly decreased ferritin expression by regulating hepcidin/FPN1 axis.

Given that hepatocytes are the primary source of hepcidin in the body [1], we utilized primary hepatocyte cultures to elucidate this regulatory mechanism. Previous studies have confirmed that CB2R is widely expressed in liver non-parenchymal cells such as Kupffer cells, hepatic stellate cells, and hepatic endothelial cells [44, 45]. In recent years, more and more studies have detected the expression of CB2R in hepatocytes, although its content is significantly lower than that in non-parenchymal cells [9, 21, 22]. Immunofluorescence localization showed that CB2R was expressed in mouse hepatocytes and Kupffer cells. The transcription of the gene encoding hepcidin gene is mainly regulated by the BMP/SMAD pathway as well as the inflammation-regulated IL-6/STAT3 pathway [20, 46]. Our data showed that CB2R activation inhibits STAT3 phosphorylation, while CB2R deficiency enhances p-STAT3 level without significantly altering SMAD phosphorylation, indicate that CB2R primarily restrains hepcidin via the IL-6/STAT3 pathway rather than the BMP/SMAD pathway in hepatocytes. CB2R is mainly coupled to the inhibitory G protein (Gi/o) [47]. Its activation inhibits adenylate cyclization (AC) and reduces intracellular cyclic adenosine monophosphate (cAMP) levels, thereby reducing protein kinase A (PKA) activity [48]. PKA has been shown to positively regulate STAT3 phosphorylation [49, 50]. This may be a possible mechanism by which CB2R regulates STAT3 signaling pathway. For final confirmation, future studies can be considered to detect the changes in cAMP/PKA activity after CB2R activation, or to observe whether the inhibitory effect of JWH133 on p-STAT3 can be reversed by using PKA inhibitors.

Macrophages are also indispensable for systemic iron metabolism [26, 51]. Macrophages can extract iron and recycle it for further use by engulfing senescent or damaged red blood cells [52, 53]. Kupffer cells were the predominant macrophages in the liver [54, 55]. In addition to the hepatocyte hepcidin mechanism, we investigated alternative pathways of iron regulation in Kupffer cells. We found that CB2R regulated FPN1 transcription. It has been confirmed that FPN transcription can be activated by Nrf2 and inhibited by transcription factors (BTB and CNC homology 1, BACH1) [29, 56, 57]. Nrf2 activation increases FPN1 expression by binding antioxidant-response elements (AREs) within the FPN1 promoter [57]. Therefore, the detection of Nrf2 showed that CB2R activation promoted Nrf2 protein expression, while CB2R-KO inhibited Nrf2 protein expression. These results supported that CB2R activation in iron overload of Kupffer cells may promote FPN1 transcription by regulating Nrf2 signaling to promote iron efflux from cells.

**Fig. 7:**
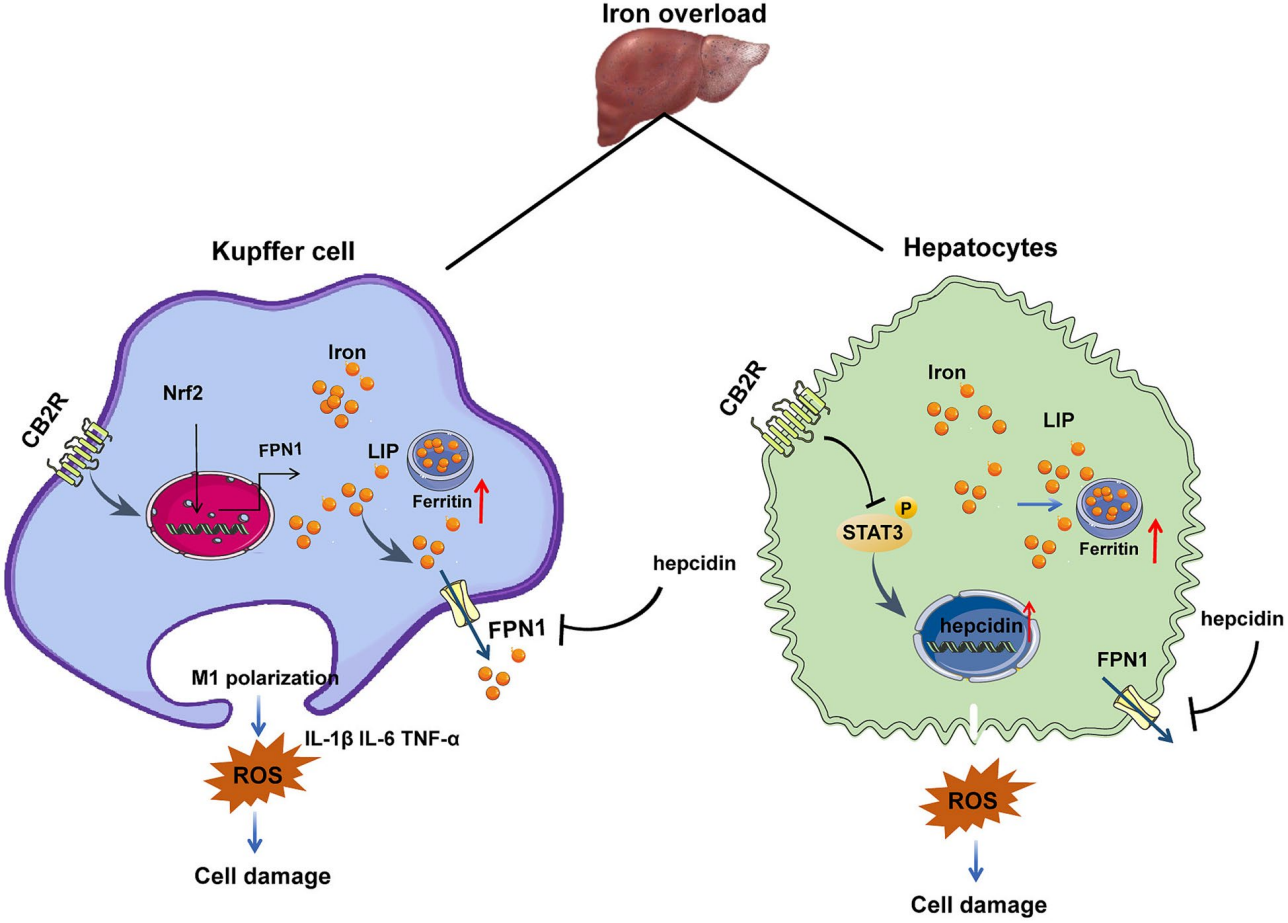
Schematic diagram of CB2R-mediated hepatic iron homeostasis. CB2R regulates iron metabolism in hepatocytes and kupffer cells. Iron overload leads to excessive iron deposition in the liver. In hepatocytes, excess iron increases the labile iron pool and ferritin, activates STAT3, and induces hepcidin production, which in turn down-regulates FPN1, exacerbating intracellular iron retention, ROS production, and hepatocyte injury. In Kupffer cells, iron accumulation similarly expands the labile iron pool, promotes M1 polarization and the release of IL-1β, IL-6, and TNF-α, amplifications oxidative stress and inflammatory damage, and drives stellate cell-mediated fibrosis. CB2R activation counteracts these processes by cell type-specific mechanisms: in hepatocytes, CB2R inhibits STAT3-dependent hepcidin induction to preserve FPN1-mediated iron efflux, whereas in Kupffer cells, CB2R enhances Nrf2-driven FPN1 expression, thereby reducing LIP and ROS production. In summary, CB2R signaling coordinates the responses of hepatocytes and macrophages to restore hepatic iron homeostasis and limit liver injury caused by iron overload

This study identified two cell type-specific pathways through which CB2R regulated iron metabolism: STAT3/hepcidin pathway in hepatocytes and Nrf2/FPN1 pathway in Kupffer cells (Fig. 7). Although these two pathways appear to be parallel in different cells, the regulation of iron homeostasis is a holistic process in which different cells work together to maintain iron homeostasis in the body. Inhibition of the STAT3 pathway in hepatocytes prevents the hepcidin-mediated degradation of FPN1, while the activation of the Nrf2/FPN1 pathway in Kupffer cells accelerates local iron cycling and prevent local iron overload.

Despite the significant protective effects observed, several limitations of the present study should be acknowledged. First, the iron-dextran model relies on parenteral iron administration, which does not fully recapitulate the pathophysiology of hereditary hemochromatosis or transfusional siderosis, where chronic iron loading and intestinal absorption are dysregulated. Second, while our focus on the Nrf2/FPN1 pathway in Kupffer cells was heavily informed by established literature, the absence of unbiased transcriptomic or proteomic screening restricts our capacity to definitively rule out the involvement of other parallel downstream pathways. Third, other vital proteins involved in iron metabolism, such as transferrin receptor 1 (TfR1) and DMT1, were not evaluated in the current experimental scope and warrant comprehensive investigation. Finally, future studies employing cell type-specific CB2R-KO or overexpression models are needed to better delineate the complex crosstalk between hepatocytes and Kupffer cells. Addressing these questions in future experiments will further optimize therapeutic strategies targeting CB2R for iron overload-related diseases.

In conclusion, our findings suggest that CB2R can act as a protective factor for the liver to neutralize iron-induced cytotoxic cascades, while playing a key role in maintaining hepatic iron homeostasis by regulating hepatocyte hepcidin signaling and macrophage iron recycling. Targeting CB2R may provide a new therapeutic strategy for iron overload-related diseases.

## Materials and methods

### Materials

Reagents: Iron-dextran (D8517), Type Ⅳ collagenase (C4-22-1G), Rhodamine123 (83,702), 2′,7′-dichlorodihydrofluorescein diacetate (D6883) were purchased from Sigma (St. Louis, MO, USA). CB2R agonist JWH133 (B7941) was obtained from APExBIO (APExBIO, Houston, USA). CB2R antagonist AM630 (T14204) were purchased from TargetMol Chemicals Inc (shanghai, China). Modified H&E Stain Kit (G1121) was purchased from Beijing Solarbio Science & Technology Co.,Ltd. (Beijing, China). Prussian blue dye solution (G1029) was purchased from Servicebio (Hubei, China). Percoll separation liquid was purchased from Yeasen (Shanghai, China). Calcein acetoxymethyl ester (GC34061) was purchased from GLPBIO (California, USA).

#### Antibodies

Antibodies for GAPDH (#8884), F4/80 (#30,325), Nrf2 (#12,721), Phospho-SMAD1/5/9 (#13,820), SMAD (#9743), along with the HRP-conjugated anti-rabbit IgG (#7074), were sourced from Cell Signaling Technology (Massachusetts, USA). FTL (A5901) was from Selleck (Houston, TX, USA). Albumin (ab207327), HO-1(ab13243) and Ferritin Heavy Chain (AB65080) were from Abcam (CA, USA). Antibodies for β-actin (20,536–1-AP) and DMT1 (20,507–1-AP) were from Proteintech (Wuhan, China). Antibody for FPN1 (AB-2340981) was from alomone labs (Jerusalem, Israel). Antibodies for CB2R (BS72043) was from Bioworld Biotech (Jiangsu, China). Goat anti-Rabbit IgG Alexa Fluor™ 488 (#A-11008) was from ThermoFisher Scientific (Massachusetts, USA). Phospho-STAT3 (310,019), STAT3 (R22785) were from zen-bioscience (Chengdu, China).

#### Biochemical detections

The MDA kit (BC0025), SOD kit (BC5165), ALT kit (BC1555), AST kit (BC1565) and serum iron kit (BC1735) kit were purchased from Beijing Solarbio Science & Technology Co.,Ltd. (Beijing, China). The GSH kit was purchased from Nanjing Jiancheng Bioengineering Institute (Nanjing, China). The iron assay kit (ab83366) and mouse serum hepcidin elisa kit (ab285280) were purchased from Abcam (Cambridge, MA, USA). The mouse serum Ferritin elisa kit (41-FERMS-E01) was purchased from ALPCO (Beijing, China).

### Mice preparation

Male C57BL/6J wild-type (WT) mice (20–22 g, 8 weeks of age) were procured from Beijing Vital River Laboratory Animal Technology Co., Ltd (Beijing, China). Zimmer strain CB2R-KO mice (C57BL/6J genetic background) were donated by the Barrow Neurological Institute [58]. All animals were maintained under specific pathogen-free conditions, featuring a standard 12-h (h) light/dark circadian cycle, controlled temperature (24 ± 1 °C), and 50% ± 5% humidity, with ad libitum access to standard rodent chow and sterilized water. Prior to experimental procedures, CB2R-KO mice were genotyped, utilizing WT littermates as appropriate baseline controls. All animal protocols received ethical approval from the Animal Care and Use Committee at Qingdao University (Approval ID: QDU-AEC-2024452).

Following a 7-day acclimatization period, hepatic iron overload was induced via daily intraperitoneal (i.p.) administration of iron-dextran (100 mg/kg) over 14 consecutive days. To determine the effect of CB2R activation on iron overload, C57BL/6J male mice were randomly divided into five groups (*n* = 6 per group): Control; Iron-dextran; Iron-dextran + JWH133; JWH133; Iron-dextran + JWH133 + AM630. JWH133 and AM630 were prepared in a vehicle comprising saline with 1% DMSO and 1% Tween-80. All mice were subjected to the experiment after one week of acclimatization. Starting from the second week, mice were intraperitoneally injected with JWH133 (3mg/kg/day) or the vehicle solution, 1 h before the injection of iron-dextran. AM630 (3mg/kg/day) or the vehicle solution was injected 30 min before JWH133. The doses of Iron-dextran or JWH133 or AM630 were chosen according to previous literature [22, 24]. Furthermore, male C57BL/6 WT mice were subjected to forced expression of CB2R using recombinant AAV via tail vein (1 × 10^11^ v.g./mouse, 100 μl). The control group was given an injection of an equal volume of empty AAV. One week after the injection, the mice were randomly divided into groups (*n* = 6 per group): AAV-empty Control; AAV-empty iron-dextran; AAV-CB2R Control; AAV-CB2R iron-dextran. Element sequence of vectors: pAAV-ITR-CMV-EGFP-Cnr2-3flag-WPRE-SV40_polyA-ITR; pAAV-ITR-CMV-EGFP-MCS-3flag-WPRE-SV40_polyA-ITR. The AAV-CB2R (AAV9) and control AAV-empty (AAV9) were purchased from SyngenTech (Beijing, China). In order to further evaluate the role and mechanism of CB2R in iron-induced liver injury, we randomly divided the WT and CB2R-KO mice from the same litter into four groups (*n* = 4–6 per group): WT Control; WT Iron-dextran; CB2R-KO Control; CB2R-KO Iron-dextran.

### Histopathological assessment and Staining

Extracted hepatic tissues were immediately fixed in 4% paraformaldehyde, dehydrated through ascending ethanol series, and subsequently embedded in paraffin blocks. These blocks were sectioned at a 4 μm thickness. Following deparaffinization and rehydration, the specimens underwent standard H&E or Masson staining to evaluate architectural damage and fibrosis.

To assess hepatic iron deposition, deparaffinized sections were stained using the Prussian blue kit: incubated with iron stain for 60 min, washed thoroughly, and counterstained with nuclear dye for 1 min. After dehydration and clearing, sections were mounted in neutral resin. Trivalent iron (blue precipitates) and overall histopathology were imaged using an Olympus slide scanner (VS-ASW 2.9, Tokyo, Japan).

### Biochemical and ELISA assays

Blood samples, collected via retro-orbital bleeding under isoflurane anesthesia, were centrifuged (1500g, 10 min) to isolate serum. Serum levels of ALT and AST, alongside hepatic lipid peroxidation markers (MDA, SOD, and GSH), were quantified utilizing specific colorimetric assay kits according to the supplier’s respective manuals. Total iron concentrations in both liver homogenates and cellular lysates were spectrophotometrically measured at 593 nm using a commercial Iron Assay Kit. Additionally, serum concentrations of ferritin and hepcidin were determined via standard ELISA protocols, with optical densities recorded on a microplate reader (Leidu R-T-200C, Shenzhen, China).

### Immunofluorescent staining

For immunofluorescent staining, frozen liver sections (or fixed cells on coverslips) were permeabilized and blocked using PBS containing 0.2% Triton X-100 and 5% normal goat serum for 60 min. Samples were subsequently probed overnight at 4 °C with primary antibodies against F4/80 (1:100) or CB2R (1:200). After PBS washes, an Alexa Fluor™ 488-conjugated goat anti-rabbit secondary antibody (1:1000) was applied for 2 h at ambient temperature. Nuclei were labeled with DAPI prior to mounting. Fluorescent signals were visualized and documented utilizing the Olympus microscopy system (VS-ASW 2.9, Olympus Co., Tokyo, Japan).

### Mouse primary hepatocyte and Kupffer cell isolation and culture

Primary hepatocytes and Kupffer cells were harvested from mouse livers by applying a two-step perfusion protocol with collagenase type IV, which had been established earlier [59]. After digestion, the hepatocyte fraction was enriched through centrifugation over a 40% Percoll layer, whereas the Kupffer cells were recovered from the non-parenchymal pool by means of a discontinuous Percoll density step gradient (25%/50%). Each cell population was then seeded individually onto culture plates precoated with collagen and cultivated in DMEM supplemented with standard nutrients. Hepatocytes received an incubation period of 24 h to allow firm adhesion, while Kupffer cells were gently rinsed at 4 h post‑seeding to eliminate floating contaminants, after which the culture medium was exchanged before commencing any treatment.

### Cell treatments

Primary hepatocytes and Kupffer cells were seeded into 96-well plates, 24-well plates or 12-well plates, allowed to adhere and then treated with various drugs at the indicated doses. The dose of FAC was chosen based on our experimental results. The doses of JWH133 and AM630 were chosen according to previous literature [60, 61]. Primary hepatocytes and Kupffer cells were treated with 1 μM AM630 for 30 min followed by the addition of 1 μM JWH133, followed by the addition of 200 μM FAC 1 h later, and co-treated for 24 h before being used for experiments. In CB2R-KO primary hepatocytes and Kupffer cells were treated with 500 μM FAC for 24 h before being used for experiments.

### Measurement of the cell viability by CCK-8

After the different treatments, 100 µl of culture medium and 10 µl of CCK-8 dye were added to each well and incubated for 2 h at 37 °C. Cell viability was measured at 450 nm by using a microplate reader (Leidu R-T-200C, Shenzhen, China).

### Measurement of the LIP

To evaluate the LIP, experimental cells were loaded with 0.05 μM calcein-AM and incubated for 30 min at 37 °C, mirroring established methodologies [42]. LIP variations were quantified by analyzing the quenching of mean cellular fluorescence via flow cytometry (BD Biosciences, USA).

### Measurement of MMP and ROS

Cellular levels of MMP and ROS were respectively detected by using Rhodamine 123 and 2′,7′-dichlorodihydrofluorescein diacetate (H2DCF-DA). The cells culture medium was removed and rhodamine 123 (5 µM) or H2DCF-DA (5 µM) was added to incubate respectively for 30 min at 37 °C in the dark. Fluorescence intensity was determined by flow cytometry (Becton Dickinson, USA) under excitation and emission wavelengths of 488 and 525 nm, respectively.

### qRT-PCR and Western blot analysis

Total cellular or tissue RNA was extracted using TRIzol reagent (Invitrogen, 15596026CN, CA, US) and spectrophotometrically normalized to a concentration of 1 μg/μL. cDNA synthesis was executed utilizing a Reverse Transcription Kit (Vazyme, R323-01, Jiangsu, CN) strictly adhering to the manufacturer’s protocol. Transcriptional changes were quantified applying the 2^−△△Ct^ algorithm, normalizing target gene signals against endogenous GAPDH. Primer sequences are detailed in Table S1.

Western blot was performed as described previously [62], using primary antibodies against FPN1, FTL, FTH, pSTAT3, STAT3, pSMAD, SMAD, and Nrf2. Primary antibodies were diluted at 1:1000. HRP conjugated secondary goat anti-rabbit and goat anti-mouse antibodies were diluted at 1:10,000. Band densities were visualized and analyzed accordingly.

### Statistical analysis

The data were expressed as mean ± standard deviation (SD). All data were analyzed by GraphPad Prism 9 software (GraphPad Software Inc., San Diego, USA). The *unpaired t-test* was performed for comparisons between two groups, one-way analysis of variance (ANOVA) for comparisons between multiple groups, and *Tukey*’*s post-hoc test* for pairwise comparisons. *P* < 0.05 indicated a statistically significant difference.

## Supplementary Information

Supplementary Material 1.

## Data Availability

All data necessary for confirming the conclusions are included in this article. The datasets generated and/or analyzed during the current study are available from the author upon reasonable request.
