# Supplementary material for: Activating Cannabinoid receptor 2 alleviates iron overload-induced liver damage via dual modulation of STAT3/hepcidin and Nrf2/FPN1 pathways
Source: Mol Biomed. 2026 Aug 10;7:135. doi: 10.1186/s43556-026-00541-1 (PMC13454076; doi:10.1186/s43556-026-00541-1)
Supplement: Supplementary file 1 [file 43556_2026_541_MOESM1_ESM.docx]

**Activating Cannabinoid Receptor 2 Alleviates Iron Overload-Induced Liver Damage via Dual Modulation of STAT3/Hepcidin and Nrf2/FPN1 Pathways**

*Han Deng^1^, Yuqian Ren^1^, Yunjie Sui^1^, ZeGang Ma^1,2*^*

1. Department of Physiology, School of Basic Medicine, Qingdao University, Qingdao, China

2. Institute of Brain Science and Disorders, Qingdao University, Qingdao, China

* Corresponding author.

Corresponding author: prof. *ZeGang Ma*

Department of Physiology, School of Basic Medicine, Qingdao University,

Room507, Boya building, 308 Ningxia road, Qingdao, China, 266071

Email: mazegang@qdu.edu.cn.

**Table S1** Primers used in this study

Gene primer sequences.

| Gene | Sequence |
| --- | --- |
| TNF-α | F: 5′-GCCCAGACCCTCACACTC-3′ |
|  | R: 5′-CCACTCCAGCTGCTCCTCTI-3′ |
| IL-6 | F: 5′-CCAGTTGCCTTCTTGGGACTG-3′ |
|  | R: 5′-CAGAGAACATGTGTAATTAAGCCTCC-3′ |
| IL-1β | F: 5′-GATGATAACCTGCTGGTGTGTGA-3′ |
|  | R: 5′-GTTGTTCATCTCGGAGCCTGTAG-3′ |
| GAPDH | F: 5′-GACCTCAACTACATGGTCTACA-3′ |
|  | R: 5′-ACTCCACGACATACTCAGCAC-3′ |
| HAMP | F: 5′-GCACCACCTATCTCCATCAAC-3′ |
|  | R: 5′-TTCTTCCCCGTGCAAAGG-3′ |

F: forward, R: reverse.
